# Effects of Different Factors on Water Flow and Solute Transport Investigated by Time Domain Reflectometry in Sandy Clay Loam Field Soil

**DOI:** 10.1007/s11270-012-1246-x

**Published:** 2012-08-24

**Authors:** Hasan Merdun

**Affiliations:** Faculty of Engineering, Department of Environmental Engineering, Akdeniz University, 07058 Antalya, Turkey

**Keywords:** Vadose zone, Preferential flow, Soil properties, Application rate, HYDRUS-1D, VS2DTI

## Abstract

Factors affecting preferential flow and transport in the vadose zone need to be investigated by experiments and simulations to protect groundwater against surface applied chemicals. The objectives of this study were to investigate the effects of several factors (soil structure, initial soil water content (SWC), and application rate) and their interactions on the extent of preferential flow and transport in a sandy clay loam field soil using the time domain reflectometry (TDR) for measuring SWC and electrical conductivity (EC) in 12 treatments, modeling (by HYDRUS-1D and VS2DTI) the measured SWC and EC, and conducting statistical tests for comparing the means of the measured and modeled SWC and EC and solute transport parameters (pore water velocity and dispersion coefficient) obtained by inversely fitting in the CXTFIT program. The study results showed that the applied solution moved faster in the undisturbed, wet initial SWC, and higher application rate experimental conditions than in the disturbed, dry initial SWC, and lower application rate, respectively, based on the analysis of the changes in TDR measured SWC and EC with depth at 1, 2, 5, and 15 h of the experiments. However, the effects of interactive factors or treatments on water flow and solute transport were not clear enough. The modeling results showed that HYDRUS-1D was better than VS2DTI in the estimation of EC and especially SWC, but overall the models had relatively low performances in the simulations. Statistical test results also showed that the treatments had different flow and transport characteristics because they were divided into different groups in terms of the means of SWC and EC and solute transport parameters. These results suggest that similar experiments with more distinct interactions and modeling studies with different approaches need to be considered for better understanding the complex flow and transport processes in the vadose zone.

## Introduction

The better understanding of the mechanisms of water and solute movement through the unsaturated or vadose zone has a vital importance for managing soil and groundwater resources. The vadose zone located above the saturated zone or groundwater is a pathway for the surface-applied chemicals. Moreover, flow and transport processes in the vadose zone are much more complex compared to the saturated zone due to the spatial variability of soil properties. Therefore, field determined soil hydraulic and chemical transport properties can be useful for the protection of soil and groundwater resources from the surface-applied chemicals.

Preferential flow and transport is a serious problem in accurate predictions of contaminant transport in soils. In preferential flow less resistant pathways such as cracks, plant roots, animal holes, and natural pipes (Beven and Germann 1982; Pontedeiro et al. 2010) cause water and solutes to move through these large openings and bypassing soil matrix (White 1985; Quisenberry et al. 1994; Flury et al. 1995; Coppola et al. 2009). In other words, infiltrating water does not have sufficient time to equilibrate with slowly moving resident water in the soil matrix (Jarvis 1998). The transport of water and solutes in the vadose zone is influenced by the degree of preferential flow which is dependent upon several factors like soil texture, structure, initial soil water content (SWC), application rate, and the others (Bouma 1991; Quisenberry et al. 1993; Jarvis and Dubus 2006). The measurements of SWC and electrical conductivity (EC) in clay and silty soils have some problems because the water bounded to soil particles has different dielectric properties from free water and this water is invisible to time domain reflectometry (TDR) measurements, thus, SWC is underestimated (Persson et al. 2000). The effects of soil structure on preferential flow and transport have been investigated by several researchers (Ghodrati and Jury 1990; Flury et al. 1994; Quisenberry et al. 1994; Hangen et al. 2002; Akhtar et al. 2011). Researchers have different ideas on the effects of initial SWC on the degree of preferential flow and transport (Shipitalo et al. 1990; Edwards et al.^1992^; Ahuja et al. 1993; Kung et al. 2000a, b; Katterer et al. 2001; Hardie et al. 2011). The higher the application rate was, the more preferential flow was observed (Quisenberry et al. 1994; Hawke et al. 2006).

The development of numerical models and their applications to soil–water management are crucial for understanding the complex mechanisms and controlling factors of water flow and solute transport in soils (Merdun and Quisenberry 2004; Mooney and Morris 2008). The complexity associated with flow and transport mechanisms in especially non-equilibrium flow conditions necessitates the further research on the application of the models. The modeling approaches for preferential flow and transport in the vadose zone range from relatively simplistic models to more complex physically based dual-porosity, dual-permeability, multi-porosity, and multi-permeability models (van Genuchten and Wierenga 1976; Gerke and van Genuchten 1993; Hutson and Wagenet 1995; Jarvis 1998). The dual-porosity and dual-permeability models assume that the total soil porosity is divided into two regions, macropore and micropores. In a dual-porosity model, water is assumed to be stagnant in the matrix, but water flows through both regions of a dual-permeability model. Besides, a dual-permeability model requires the exchange of water and solutes between the matrix and the fractures (Simunek et al. 2003). Soil hydraulic and chemical transport properties are required by models to predict water and solute transport to groundwater. Recently, TDR has been widely used in the reliable measurements of water and solute transport parameters in laboratory and field conditions (Mallants et al. 1996; Comegna et al. 1999; Vogeler et al. 2001; Regalado et al. 2003; Coppola et al. 2011).

The traditional measurement techniques of flow and transport parameters in the vadose zone include the soil sampling, the use of suction cups, or collecting the leachate at the bottom of a column and measuring the concentration of leachate or solute, and then presenting the results as a breakthrough curve (BTC). Overall these techniques are tedious, destructive, time and energy consuming, and non-repeatable of the transport parameters. Unlike these techniques, TDR, which is a relatively new technique, is reliable, robust, automated, and can be used across a wide range of spatial and temporal scales, thereby getting rid of these problems. Over the last three decades, the TDR technique has been used to monitor not only volumetric SWC (*θ*), but also the bulk soil EC (*σ*). This technique is increasingly used by researchers because it is one of the easiest and most reliable ways to measure both *θ* and *σ* continuously in laboratory or field conditions. Furthermore, the accurate data collected by TDR can be used to test flow and transport models and/or characterize the parameters of these models (Vogeler et al. 1998; Lee et al. 2001; Vogeler et al. 2005).

The effects of individual factors like soil texture, structure, initial SWC, and application rate on preferential flow have been studied extensively under laboratory conditions using TDR (Vanclooster et al. 1995; Comegna et al. 1999; Vogeler et al. 2000; Gaur et al. 2003; Graeff et al. 2010), but the effects of these factors on preferential flow and transport in especially sandy clay loam field soil have not been studied collectively. Moreover, as far as my knowledge, the interactive effects of these factors on preferential flow have not been studied using TDR. Furthermore, the applications of the various developed models are relatively limited in the predictions of flow and transport parameters in especially heterogeneous field soils, where preferential flow is widely reported. Such modeling studies can be useful in better understanding of the extent and mechanisms of preferential flow in such a sandy clay loam field soil.

Therefore, the objectives of this study are to investigate the individual and interactive effects of factors like soil structure, initial SWC, and application rate on the extent of preferential flow and transport in a sandy clay loam field soil by means of TDR measured SWC and EC; simulation of the measured SWC and EC through HYDRUS-1D and VS2DTI; and comparison of the means of the measured and modeled SWC and EC, and solute transport parameters of 12 treatments using statistical analyses.

## Materials and Methods

### Experimental Area

Experiments were conducted on a sandy clay loam field soil around 10 km far from Kahramanmaraş City center in Turkey (31°55′61″ E, 41°55′39″ N) during a period of September 18 and October 29, 2007. The area was not planted in the last a few years, but there were some weeds and grasses naturally grown in the area. The experimental area was around 200 km far from the Mediterranean sea in the south with an altitude of 560 m. The area is under the Mediterranean climate with a characteristic of hot and dry summer and warm and rainy winter. The area had a mean annual temperature of 16.3 °C with the mean highest temperature of 27.9 °C and a mean rainfall of 708.1 mm. The mean highest evaporation was 333.3 mm. The highest and lowest rainfalls were observed in January and July, respectively. The studied soil has been developed on alluvial materials which are classified as Fluvent according to soil taxonomy (Gündoğan 1998). Soils with sandy clay loam have well drainage. The detailed properties of experimental soil are presented in Table 1.

**Table 1:** Some physical and chemical properties of experimental field soil

Depth (cm)	Sand (%)	Silt (%)	Clay (%)	Texture	BD (g cm^−3^)	*P* (%)	OM (%)	*K*_s_ (cm h^−1^)	pH	EC_25_ (dS m^−1^)	CaCO_3_ (%)
10	51	16	33	SCL	1.22	54	1.44	14.565	8.19	0.905	15.36
20	55	19	26	SCL	1.54	42	1.28	0.573	8.13	0.786	18.75
30	61	16	23	SCL	1.71	36	0.98	0.275	8.07	0.725	19.53
40	69	19	12	SL	1.74	34	0.78	0.076	8.01	0.603	21.35
50	82	8	10	LS	1.64	38	0.92	2.556	8.04	0.454	20.70
60	92	2	6	S	1.71	35	0.90	2.849	8.01	0.314	16.53
75	93	1	6	S	1.75	34	0.70	31.274	7.99	0.239	14.45

*SCL* sandy clay loam, *SL* sandy loam, *LS* loamy sand, *S* sand, *BD* bulk density, *P* porosity, *OM* organic matter content, *K*_s_ saturated hydraulic conductivity, *EC*_*25*_ electrical conductivity at 25 °C, *CaCO*_*3*_ calcium carbonate content

### Determination of Soil Properties

Disturbed and undisturbed soil samples were obtained from seven depths of the soil profile with three replicates to determine some physical and chemical properties (Table 1) of the experimental field soil before beginning the experiments. The undisturbed soil samples were used for the analyses of bulk density and saturated hydraulic conductivity, but the disturbed soil samples were used for the other analyses. All analyses of the samples were done by following the procedures of the “Methods of Soil Analysis, Parts 3 & 4: Chemical and Physical Methods”. The standard 100 cm^3^ cylinders were used in the determination of bulk density. Saturated hydraulic conductivity was determined on undisturbed soil samples with three replicates by usig the “Constant Head Permeameter” method. The disturbed soil samples taken from seven depths of the soil profile with three replicates were used for the other analyses. After getting the saturation extracts from the sieved samples in a 2-mm sieve EC, pH, and temperature of the extracts were measured by using the EC/pH/T meter. EC_25_ values were calculated after correcting the measured EC values at 25 °C. Organic matter contents of the samples were calculated by multiplying the organic carbon contents by 1.724. Texture analyses of the samples were done using the “Hydrometer Method”.

### Solution Application Rates

Salt-solution (CaCl_2_) application rates of rainfall simulator; 1.5 m by 1.0 m by 0.30 m in dimensions, made of an aluminum sheet with the thickness of 1 mm, and having 150 injectors (10 by 15 with 10 cm space) at the bottom of its tank located horizontally 20 cm above the plot surface; were determined before beginning the experiments. Firstly, the solution concentration to be applied was determined as 3,200 mg/L based on the current soil EC, the applied water EC, and the EC sensing capacity (4.5 dS/m) of the TDR probes. Then the simulator application rates were determined for every 1 cm solution heads (from 6 cm up to 15 cm heads) in the simulator tank. To do that, after adding 6 cm solution head into the simulator the solution passing through the injectors was collected through a plastic sheet laid down the simulator. The 3-l leachates were collected several times during 1.5–2 h. For 6 cm solution head the mean solution application rate was determined by averaging 3 l leachates. The mean solution application rates were determined for the other solution heads in a similar way. The depth of solution in the simulator was kept constant as much as possible to get a constant solution application rate by adding the equal amount of solution flowing out the simulator every 1 h when the solution head was 6, 7, and 8 cm; every 40 min in 9 and 10 cm heads; and every 30 min in the other heads.

### Experimental Treatments

Three main factors and several sub-factors (in parenthesis): soil structure (disturbed and undisturbed), initial SWC (dry and wet), and solution application rate (low, intermediate, and high) with their interactions resulted in 12 (2 × 2 × 3) experimental tretaments (Table 2). A total of 24 (2 × 2 ×3 × 2) plots were used in this experimental study because the experiments were designed based on the factorial block design with 2 replicates.

**Table 2:** Experimental treatments

Treatment	Soil type	Soil structure		Initial soil water content		Solution application rate		
	SCL	Undisturbed	Disturbed	Dry	Wet	Low	Intermediate	High
1	X	X		X		X		
2	X	X		X			X	
3	X	X		X				X
4	X		X	X		X		
5	X		X	X			X	
6	X		X	X				X
7	X	X			X	X		
8	X	X			X		X	
9	X	X			X			X
10	X		X		X	X		
11	X		X		X		X	
12	X		X		X			X

*SCL* sandy clay loam

The undisturbed treatment was obtained by keeping the natural condition of field soil. The disturbed treatment was formed by digging the surface soil (1.5 × 1.0 m) of the plot till 20 cm depth, sieving in a 10-mm sieve, and then replacing its original place. The dry initial SWC treatment was produced by keeping the natural soil water condition which was close to the permanent wilting point. The wet initial SWC treatment was obtained after uniformly applying 10 cm of water to the plot surface before the expriment by using a hand-held plastic filtered container. The plot surface was covered by a plastic sheet to prevent soil water loss through evaporation and then waiting for 2 days for the initial SWC to reach about the field capacity. The low and intermediate solution application rates were determined so that no ponding occured on the soil surface during the application of the constant amount (12 cm) of solution using the rainfall simulator. The treatment of high solution application rate was obtained by, firstly, inserting the metal frame (1.5 × 1.0 × 0.25 m and the thickness of 1 mm) carefully into the soil around 5 cm to prevent lateral flow of the applied solution. Then a plastic sheet (2 × 2 m) was laid down the bottom surface of the metal frame and 12 cm CaCl_2_ solution was poured on the sheet. Finally, the solution was rapidly applied to the soil surface by cutting the plastic sheet.

### Principles of Time Domain Reflectometry

Campbell Scientific TDR100 was used for all experiments (Campbell Scientific, Logan, UT). A TDR system mainly consists of a cable tester or TDR itself, coaxial connection cable, multiplexers, TDR probes, datalogger, laptop computer, and the software which controls the TDR measurements. For automated measurements the TDR can be controlled via a datalogger, otherwise, via a computer with Windows software (PCTDR). For automatic control a datalogger with the TDR and multiplexers is needed.

The TDR device sends an electromagnetic pulse which travels through the coaxial cable and the TDR probe embedded in the soil. The TDR device measures the propagation and reflection (at the end of the probe) of the pulse, and its attenuation. The attenuation of the pulse depends on the dielectric constant (*ε*) of the soil, water, and air. The dielectric constants of liquid water, soil minerals, and air are 80, 3–5, and 1, respectively, at a frequency of 1 GHz and a temperature of 20 °C (Weast 1965). The *ε* is calculated by (Topp et al. 1980) as:

1 $$ \varepsilon = {\left[ {\frac{{ct}}{{2{l_t}}}} \right]^2} = {\left[ {\frac{{{l_a}}}{{{l_t}{\nu_p}}}} \right]^2} $$

where *c* is the propagation velocity of an electromagnetic wave in free space (3 × 10^8^ m/s), *t* is the travel time (s), *l*_t_ is the real length of the transmission line (m), *l*_a_ is the apparent length (m) measured by the TDR, and *ν*_p_ is the relative velocity setting of the device. High volumetric SWC (*θ*) produces a high value of *ε*. Therefore, the ε of the soil can be related to the θ by means of the calibration equation of Topp et al. (1980) as:

2 $$ \theta { } = { } - { 5}.{\text{3 x 1}}{0^{{ - {2}}}} + { 2}.{\text{92 x 1}}{0^{{ - {2}}}}\varepsilon { } - {5}.{\text{5 x 1}}{0^{{ - {4}}}}{\varepsilon^{{2}}} + { 4}.{\text{3 x 1}}{0^{{ - {6}}}}{\varepsilon^{{3}}} $$

Equation 3 can be used for nearly all mineral soils, but specific calibrations are required for organic or other soils (Patterson and Smith 1981; Nadler et al. 1991).

Even though several procedures were developed by various researchers (Dalton et al. 1984; Zegelin et al. 1989; Nadler et al. 1991) to determine soil bulk EC from TDR waveforms, Topp et al. (1988) proposed an alternative procedure as:

3 $$ \sigma = \left( {\frac{K}{{{Z_u}}}} \right)\left( {\frac{{1 - {\rho_{\infty }}}}{{1 + {\rho_{\infty }}}}} \right) $$

where *K* is the geometric constant of a probe (m^−1^), *Z*_u_ is the characteristic impedance of a cable (Ω), and *ρ*_∞_ is the reflection coefficient. The reflection coefficient is calculated as, *ρ*_∞_ = (*V*_∞_ − *V*_0_)/*V*_0_, where *V*_∞_ is the signal amplitude at the distant point and *V*_0_ is the signal amplitude from the TDR device.

### Application of Experiments

After a soil profile (approximately 50–60 cm in width and 80–90 cm in depth) was dug along the long side of the experimental plot (1.5 × 1.0 m), 3 CS630 model TDR probes (3-rod, 15 cm long, and 0.318 cm in diameter) were horizontally inserted into soil using a probe insertion guide to measure SWC and EC in soil depths of 10, 20, 30, 40, 50, 60, and 75 cm. The total of 21 TDR probes inserted to the soil looked like a 7-level stair starting from the surface right to the bottom left of the profile. Such a configuration of the probes allowed the disturbance of an upper probe not to affect a lower probe. In addition, 7 thermocouples (one for each depth) were inserted into the soil near the probes to measure soil temperature. After the configuration of the TDR system (connecting the probe cables to multiplexers (the number of 3), and connecting multiplexers to the TDR through the CR1000 datalogger) initial SWC and EC were measured by the TDR before the solution application. Then a total of 12 cm CaCl_2_ solution (3,200 mg/L) was applied to the plot surface at a certain rate through the rainfall simulator. Drinking water from the network was used in the formation of solutions in the experiments. As soon as the application finished, the plot surface was covered by a plastic sheet in order to prevent evaporation from the plot. By assuming that flow reaches equilibrium around 1.5 days in a sandy clay loam soil, the TDR system was operated for around 1.5 days in each plot. The parameters of La/L for SWC, EC, soil temperature, and waveform were measured by the TDR for each probe every 15 min and stored in the datalogger automatically.

### Measured Data

Since the measured EC and especially SWC were unstable with time in sandy clay loam soil, the main trends of SWC and EC with time were visually determined by plotting the data and the outliers were removed. The SWC and EC values for a depth were determined by averaging three probe readings. For a specific depth the final SWC and EC values were obtained by avering the values of six probe readings because the experiments were conducted with two replicates. Besides, soil bulk EC (BEC) readings of the TDR were corrected as as EC_25_ = BEC (1 + 0.02 (25-Tsoil), where Tsoil is soil temperature (°C) around the related TDR probes measured by thermocouple because they were sensitive to solute concentration and temperature. The corrected EC data were used in the development of BTCs, the change of EC with time *t*, for each depth of each plot.

### Introduction of Models

#### HYDRUS-1D

HYDRUS-1D is a model which simulates one-dimensional movement of water, heat, and multiple solutes in the unsaturated, partially saturated, or fully saturated porous media (Simunek et al. 2005). The flow and transport domain may be non-uniform or layered soils. Physicallly non-equilibrium solute transport is considered by assuming dual-porosity approach, where the total soil porosity is partitioned into two regions, mobile and mimmobile.

While HYDRUS-1D uses the Richards Equation for one-dimenional saturated and unsaturated water flow, the convection–dispersion equation (CDE) is used for non-reactive solute transport in steady flow condition as:

4 $$ \frac{{\partial \theta }}{{\partial {\text{t}}}} = \frac{\partial }{{\partial {\text{z}}}}\left[ {{\text{K}}\left( {\text{h}} \right)\left( {\frac{{\partial {\text{h}}}}{{\partial {\text{z}}}} + 1} \right)} \right] $$

5 $$ \frac{{\partial {\text{c}}}}{{\partial {\text{t}}}} = \frac{\partial }{{\partial {\text{z}}}}\left( {{\text{D}}\frac{{\partial {\text{c}}}}{{\partial {\text{z}}}}} \right) - \nu \frac{{\partial {\text{c}}}}{{\partial {\text{z}}}} $$

where *θ* is the volumetric SWC (cm^3^ cm^−3^), *t* is the time (h), *h* is the soil water pressure (cm), *z* is the vertical coordinate or gravitational head (cm), *K*(*h*) is the unsaturated hydraulic conductivity (cm h^−1^) as a function of *h*, *c* is the solute concentration (mg L^−1^), *D* is the dispersion coefficient (cm^2^ h^−1^), and *v* is the pore water velocity (cm h^−1^).

The unsaturated soil hydraulic properties are described by several analytical functions such as Brooks and Corey (1964), van Genuchten (1980), Durner (1994), and Kosugi (1996). However, the hydraulic function of van Genuchten (1980) for soil water retention curve (SWRC) and the hydraulic conductivity function of Mualem (1976) and van Genuchten (1980) were used in this study and described as:

6 $$ {S_e}(h) = \frac{{\theta (h) - {\theta_r}}}{{{\theta_s} - {\theta_r}}} = {\left( {1 + \alpha {{\left| h \right|}^n}} \right)^{{ - m}}} $$

7 $$ {\text{K}}\left( {\text{h}} \right) = {{\text{K}}_{\text{s}}}{\text{ S}}_{\text{e}}^{\text{l}}{\left[ {{1} - {{\left( {{1} - {\text{S}}_{\text{e}}^{{{\text{1/m}}}}} \right)}^{\text{ m}}}} \right]^{{{ 2}}}} $$

where *S*_e_ is the effective saturation (dimensionless), *θ*_r_ and *θ*_s_ are the residual and saturated SWC (cm^3^ cm^−3^), respectively, *α* (cm^−1^), *m* and *n* are the shape parameters of SWRC, *K*_s_ is the saturated hydraulic conductivity (cm h^−1^), *l* is the pore connectivity, and *m* = 1 − 1/*n* when *n* > 1. HYDRUS-1D offers several options for initial and boundary conditions for water flow and solute transport. The flow and transport equations are solved numerically by the finite element method using the constitutive relations between soil hydraulic variables (*θ*, *h*, and *K*) and initial and boundary conditions.

### VS2DTI

VS2DTI is a model using the single-porosity or single-permeability or equilibrium modeling approach. In this modeling approach, it is assumed that soil pores are uniformly distributed throughout soil; therefore, flow is uniform in such a soil. VS2DTI is a graphical software package used to simulate water flow and solute transport in unsaturated porous medium (Hsieh et al. 2000). VS2DTI is a model solving the Richards Equation and the CDE for water flow and solute transport, respectively, by using the finite-difference method. The Brooks and Corey (1964), van Genuchten (1980), Haverkamp et al. (1977), and other equations are used as the soil hydraulic functions in the model. Initial hydraulic condition can be defined by static equilibrium as SWC or pressure value. Boundary conditions can be defined with different parameters such as pressure or total head, flux, infiltration under ponding, evaporation, transpiration, and seepage surfaces.

The data need for running the model are the geometry of the study area, initial SWC or pressure head, initial concentration or temperature, hydraulic and transport properties, dispersivity, and molecular diffusion. Graphical user interface (GUI) consists of pre-processor and post-processor windows. The pre-processor window is used to define soil profile, hydraulic and transport properties, initial and boundary conditions, the division of soil profile into grids, and other model parameters. Spatially variable parameters can be graphically input and edited by drawing tools as in other graphical programmes. The post-processor window can be opened to run the model after all required parameters were input to the model. The model data input files are automatically generated in the related folder as soon as the post-processor window is open. The simulation results for a given time can be shown as soil water pressure head, SWC, saturation level, temperature, concentration, and flux contours.

### CXTFIT

CXTFIT is used to determine the solute transport parameters by inversely (inverse modeling) fitting the one-dimensional CDE to the solute transport data (BTCs) obtained from the field or laboratory experiments (Toride et al. 1995). In addition, it can be used in order to estimate solute concentration with space or time directly (direct or forward modeling) by using the obtained transport parameter values. The inverse estimation procedure includes the minimization of the objective function, the sum of the squares of the differences between the measured and fitted concentrations. CXTFIT model has three different one-dimensional transport scenarios; conventional equilibrium CDE, physical or chemical non-equilibrium CDE, and stochastic stream tube model. The model includes six different concentration modes, six boundary conditions, and five initial conditions.

### Determination of Model Parameters

HYDRUS-1D and VS2DTI models were used in the simulation of SWC and EC measured by the TDR every 15 min during around 36 h in 7 depths of different profiles of field sandy clay loam soil. Thus, since different parameters were used by the models in these simulations, the determination of these parameter values is explained as follows.

In the HYDRUS-1D simulations the van Genuchten-Mualem (van Genuchten, 1980) hydraulic models were used. The values of van Genuchten (1980) hydraulic function parameters (*θ*_r_, *θ*_s_, *α*, *n*) were obtained by fitting the measured SWRC data to the function using the RETC programme (van Genuchten, M. Th et al. 1991). Then the values of Mualem (1976) model parameters (*K*_s_, *l*) were determined by using the obtained van Genuchten’s 4 parameters as input to the Rosetta programme (Schaap et al. 2001). However, the *K*_s_ values estimated by the pedotransfer functions integrated into the MACRO model (Larsbo and Jarvis 2003) were used in the modeling because the *K*_s_ values obtained by fitting the Mualem (1976) model and the measurements were unacceptably small. While the variable pressure head was selected as the top boundary condition, the constant pressure head was selected as the bottom boundary condition for water flow in the model. The equilibrium model was selected as the solute transport model. For the solute transport boundary conditions the concentration flux was selected for the top and the zero concentration gradient for the bottom. For the time variable boundary conditions two different boundary values (during the solution application and from the end of the solution application till the end of the TDR measurements) and the values needed for these parameters (the duration of solution application, application rate, concentration, the duration of no solution application during the measurements) were input to the model. After inputting the TDR measured initial SWC and EC values the model was run for the simulations.

In VS2DTI model simulations, firstly, physical or geometric dimensions and layer thicknesses of the profile of a studied experimental treatment were drawn by using the drawing tools in the model. Secondly, flow and transport model, iteration, and output options were defined in the model. Thirdly, the values of hydraulic and solute transport parameters of each layer were determined. The *K*_s_ values were determined by utilizing the pedotransfer functions integrated into MACRO model. Saturated SWC values were obtained from SWRCs. The values of soil hydraulic parameters (*θ*_r_, *θ*_s_, *α*, *n*) used in the model were determined by fitting the van Genuchten hydraulic function to SWRC data by using RETC programme (van Genuchten et al. 1991). Initial SWC and EC values were input to the model as contour value on the drawn domain. Finally, the boundary conditions were set and the domain was divided into the grids.

In the inverse modeling with CXTFIT programme the options of the stochastic equilibrium CDE model and the field-scale resident concentration were set in the model. After selecting the option of pulse input at application time *t* as the boundary condition in the model the solution concentration (3,200 mg L^−1^) and the duration of application were defined. The measured initial concentrations were input to the model as initial condition after selecting the constant initial concentration. After inputting the measured BTC data to the model and then running it the values of solute transport parameters (pore water velocity, *v*, and dispersion coefficient, *D*) were obtained.

### Model Performance Evaluation

The means of SWC and EC values measured along the profiles of the treatments and the means of the measured and estimated (by 2 models) values of SWC and EC in a treatment were compared by Tukey test using the SPSS statistical programme and then they were grouped using the letters. In addition, two statistical parameters such as the coeficient of model efficiency (CME) and the root mean square error (RMSE) were used to determine the performances of the models (HYDRUS-1D and VS2DTI) in estimation of SWC and EC at a certain depth of a treatment as:

8 $$ CME = \frac{{\sum\nolimits_{{i = 1}}^n {{{\left( {{O_i} - {O_m}} \right)}^{{{ }2}}} - \sum\nolimits_{{i = 1}}^n {{{\left( {{P_i} - {O_m}} \right)}^{{{ }2}}}} } }}{{\sum\nolimits_{{i = 1}}^n {{{\left( {{O_i} - {O_m}} \right)}^{{{ }2}}}} }} $$

9 $$ {\text{RMSE}} = \sqrt {{\frac{{{{\sum\nolimits_{{{\text{i}} = {1}}}^{\text{n}} {\left( {{{\text{O}}_{\text{i}}} - {{\text{P}}_{\text{i}}}} \right)} }^{{{ 2}}}}}}{\text{n}}}} $$

where *O* is the observed or measured, *P* is the model prediction or estimation, *O*_m_ is the mean of observed, and *n* is the sample number. The CME value ranges from −∞ to +1 and the model performance is perfect when CME is equal to 1. RMSE has values ranging from 0 to +∞ and its value becomes zero when the model perfectly simulates the measured data.

## Results and Discussion

### Soil Properties and Application Rates

Soil texture changed with depth from sandy clay loam to sandy (Table 1). Specifically, the soil had sandy clay loam texture at the surface 3 depths, sandy loam at the 4th depth, loamy sand at the 5th depth, and sandy at the last 2 depths. Since the texture was sandy clay loam in especially surface layers that controlled water and solute transport in the profile, overall the soil texture was named as sandy clay loam. Bulk density of the soil increased with depth as the sand content increased. Porosity changed with depth based on the bulk density because it was calculated by using the bulk density-particle density relation with a constant particle density (2.65 g cm^−3^ for mineral soils). Organic matter content of the soil was in intermediate level at the surface 3 depths, but it was lower and relatively constant at the last four depths. The *K*_s_ values of the soil were relatively low except in the first and last three depths, where the sand was dominant towards the bottom of the profile. The pH level of the soil was basic (higher pH) and changed with depth slightly. EC values of the soil at 25 °C decreased with depth as the clay content decreased (Table 1). These considerable changes in soil properties with depth made this soil an interesting study area.

The solution application rates were determined as the 2.263 and 3.238 cm h^−1^ for low and intermediate application rates in sandy clay loam soil by applying the same amount (12 cm) of solution within 5.303 and 3.706 h, respectively, so that no ponding occured in the soil surface. It is well-known that when solution application rate is higher than soil infiltration rate, ponding is inevitable, thus, resulting in preferential flow. For high application rate the same amount (12 cm) of solution was applied with different durations and rates according to the treatments. Ponding was unavoidably observed during the high application rate, but it was not observed for low and intermediate application rates during the experiments.

### Experiments and Modeling

**Fig. 1:**
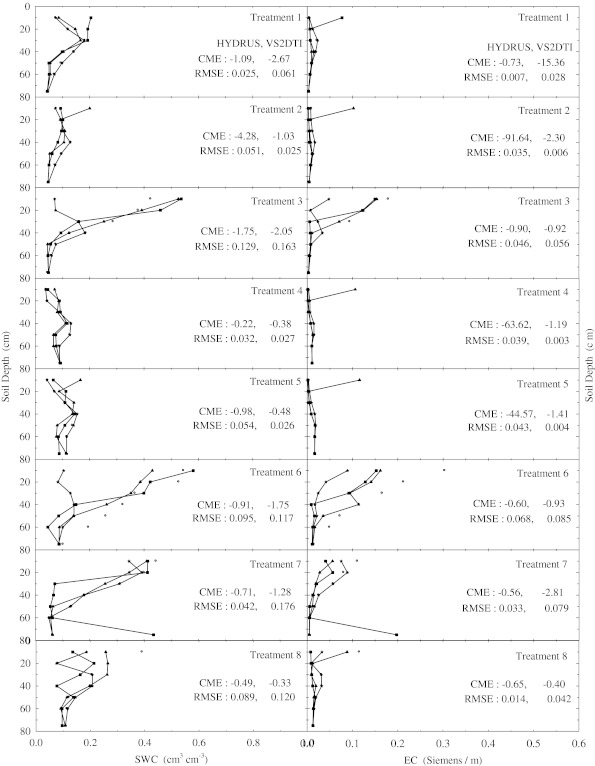
Soil water content (SWC) and electrical conductivity (EC) variation with depth at 1 h of the experiment in the eight treatments. The initial, measured, and model (HYDRUS and VS2DTI) values are defined by symbols *star*, *circle*, *filled triangle*, and *filled square*, respectively. CME and RMSE are the coeficient of model efficiency and the root mean square error, respectively

After the application of salt-solution (CaCl_2_) to each of 12 plots or treatments SWC and EC at 7 depths were measured by TDR every 15 min during around 1.5 days. However, the results of the only first eight treatments (see Table 2) at 1, 2, 5, and 15 h after the initiation of the experiments were visually presented in Fig. 1 through 4, respectively, because of the space limitation in the paper, to better investigate the extent of preferential flow and transport. On the other hand, the results of statistical anaylses for all treatments were tabulated in Tables 4 and 5. Besides, the experimental and modeling results of the remaining treatments were discussed in the text.

**Fig. 2:**
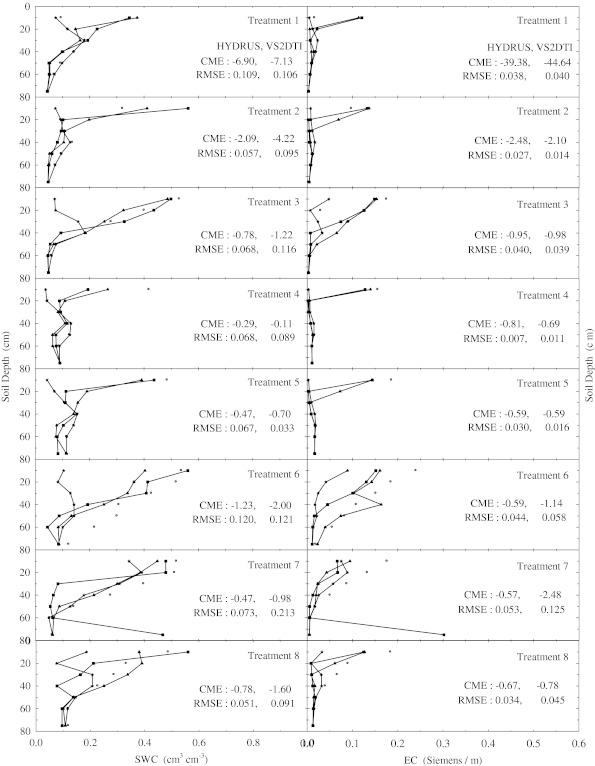
Soil water content (SWC) and electrical conductivity (EC) variation with depth at 2 h of the experiment in the eight treatments. The initial, measured, and model (HYDRUS and VS2DTI) values are defined by symbols *star*, *circle*, *filled triangle*, and *filled square*, respectively. CME and RMSE are the coeficient of model efficiency and the root mean square error, respectively

**Fig. 3:**
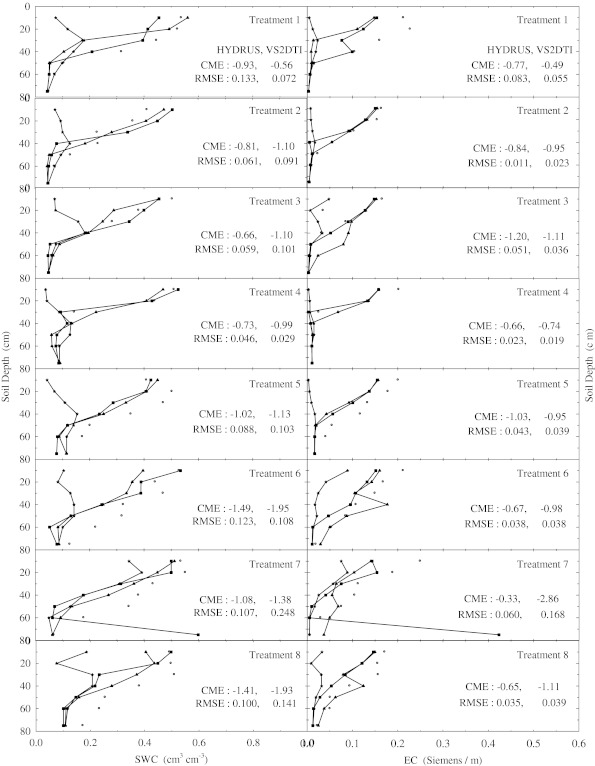
Soil water content (SWC) and electrical conductivity (EC) variation with depth at 5 h of the experiment in the eight treatments. The initial, measured, and model (HYDRUS and VS2DTI) values are defined by symbols *star*, *circle*, *filled trianlge*, and *filled square*, respectively. CME and RMSE are the coeficient of model efficiency and the root mean square error, respectively

**Fig. 4:**
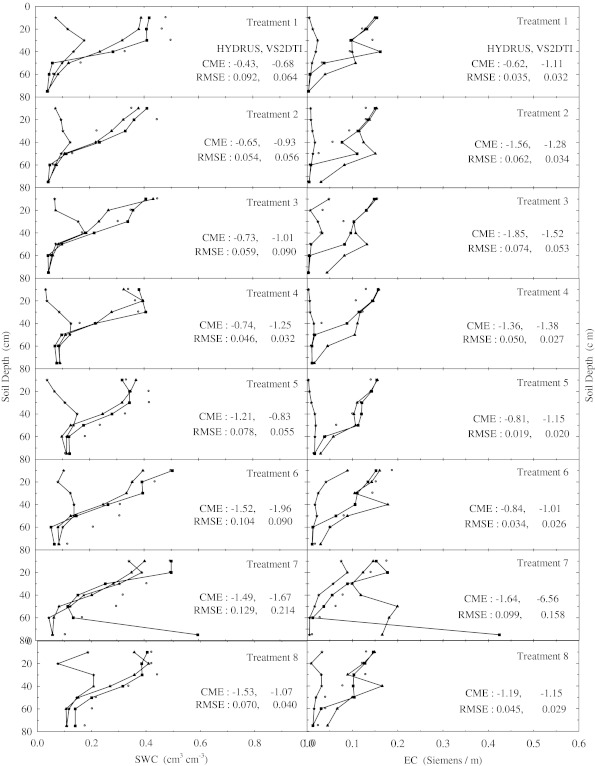
Soil water content (SWC) and electrical conductivity (EC) variation with depth at 15 h of the experiment in the eight treatments. The initial, measured, and model (HYDRUS and VS2DTI) values are defined by symbols *star*, *circle*, *filled triangle*, and *filled square*, respectively. CME and RMSE are the coeficient of model efficiency and the root mean square error, respectively

There were no changes in the SWC and EC with depth compared to the initial SWC and EC in the treatements 1, 2, 4, and 5 within the 1st hour of the experiments (Fig. 1). However, SWC and EC increased up to 20 cm in the treatments 7 and 8, 40 cm in the treatment 3, 50 cm in the treatments 10 and 11, 60 cm in the treatment 9, and 75 cm in the treatment 12. Furthermore, the solution passed the 75 cm depth or bottom of the profile of the treatment 6 just 1 h after beginning of the experiment, where the increase in SWC and EC compared to the initial values is clearly shown in Fig. 1. In the 2nd h of the experiments the applied solution increased SWC and EC up to different layers of the treatment plots except the 1st treatment, where there were no changes in the SWC and EC throughout the profile (Fig. 2). SWC and EC increased up to 20 cm in the treatments 2, 4, and 5; 40 cm in the treatment 3; 50 cm in the treatment 8; 60 cm in the treatments 7, 9, and 10; and 75 cm in the treatments 11 and 12. However, the applied solution again passed the bottom of the profile of the treatment 6 2 h after the experiment started (Fig. 2). In the 5th h of the experiments, the applied solution raised SWC and EC until 40 cm in the treatments 3 and 4; 50 cm in the treatment 1; 60 cm in the treatment 2; and 75 cm in the treatments 5, 7, 9, 10, 11, and 12 (Fig. 3). However, the solution left out the profiles of the treatments 6 and 8 as seen in Fig. 3, where SWC and EC increased significantly at the last depths (75 cm). Overall as the time passed the applied solution moved further down in the profiles of all treatments as expected (Fig. 4). The applied solution increased SWC and EC up to 40 cm in the treatment 3; 50 cm in the treatment 4; 60 cm in the treatments 1 and 2; and 75 cm in the treatments 5, 9, 10, 11, and 12 (Fig. 4). However, the solution passed down the bottom of the profiles of the treatments 6, 7, and 8 in the 15th h of the experiments.

The applied solution did make no changes in SWC and EC in the 1st h of the experiments in the treatments 1, 2, 4, and 5; but it increased them up to different depths during the 2nd, 5th, and 15th h of the experiments with similar trends between the treatments 1 and 4 and between the treatments 2 and 5. The only difference between the 1st and 4th treatments and between the 2nd and 5th treatments was that the top 20 cm of soil profiles were disturbed in the latter treatments under dry initial SWC conditions. Similarly, SWC and EC increased up to lower depths of the treatments 10 and 11 at earlier times (1 and 2 h), but the applied solution moved lower depths and passed down the profiles of the treatments 7 and 8 as the time passed. The only difference between the 7th and 10th treatments and between the 8th and 11th treatments was the disturbance of the profiles of the treatments 10 and 11 under wet initial SWC conditions. The results showed that the applied solution moved faster in the undisturbed soil and even passed out the profiles at later times compared to the disturbed soil in especially wet initial SWC conditions. Therefore, the effects of disturbed and undisturbed soil conditions on water flow and solute transport were more distinctive in wet initial SWC condition. Andreini and Steenhuis (1990) found that while preferential flow was only effective in undisturbed part of the disturbed column, it was effective in the whole part of the natural soil column. Similarly, dye tracer moved much deeper depth in structured soils than in non-structured soils (Flury et al. 1994). In addition, Kulasekera et al. (2011) confirmed the existence of the short-term preferential flow during rainfall events and the water infiltrated into the deeper depths in the no-tilled plot as opposed to the conventionally tilled plot.

The applied solution increased SWC and EC with time through the profiles of the treatments 7 and 8 more than that of the treatments 1 and 2 under undisturbed soil conditions. Similarly, the applied solution raised SWC and EC with time through the profiles of the treatments 10 and 11 more than that of the treatments 4 and 5 under disturbed soil conditions. These results showed that water and solute moved more rapidly in the wet initial SWC conditions than in the dry initial conditions. However, the effects of dry and wet initial SWC conditions on flow and transport characteristices were more obvious under the disturbed soil conditions. Several researchers reported that the higher initial SWC increased the leaching rates of water and solutes to groundwater (Ahuja et al. 1993; Kung et al. 2000a, b).

The applied solution moved lower depths in the treatments 2, 5, 8, and 11 than in the treatments 1, 4, 7, and 10, respectively; where the only difference between the 1st and 2nd group of treatments was that the 1st group of treatments had higher application rates under different soil structural and initial SWC conditions. The solution applied to the treatments 3, 6, 9, and 12 with the highest application rate or flood irrigation reached the bottom of almost all profiles except the treatment 3, where the applied solution possibly leached out the profile through macropores by bypassing the soil matrix. There seemed to be no clear interactive effects of soil structure and initial soil water content on application rates in terms of water and solute movement. Akhtar et al. (2011) reported that water moved lower depths in soil profile under higher application rates. In addition, Quisenberry et al. (1994) measured the effects of application rate on the movement through macropores and spatial distribution of water and chloride and the results showed that preferential flow was effective in the high solution application rate.

The agreements between SWC and EC in the profiles of all treatments (Figs. 1, 2, 3 and 4) were good, indicating a certain degree of preferential or non-uniform flow. The agreement means that the increase in EC corresponds to the increase in SWC. The poor agreement between them was the indication of significant displacement of the antecedent soil solution, resulting in the piston-type or uniform flow. If the applied solution moved by complete displacement or piston-type flow, it would be able to reach about 27.30 cm in the 1st treatment and about 23.90 cm in the 5th treatment.

Two statistical parameters (CME and RMSE) were calculated to evaluate the simulation performances of 2 models (HYDRUS and VS2DTI) in the estimation of SWC and EC and the results were presented on Figs. 1, 2, 3, and 4. Even though both parameter values were presented on Figs. 1, 2, 3, and 4, only CME values were used in the discussion of the model performances in the text because CME had the ability to evaluate both the comparative and absolute performance of a given model. VS2DTI uses only single-porosity, or single-permeability, or equilibrium modeling approach, but HYDRUS-1D uses both single-porosity and dual-porosity or non-uniform modeling approaches. However, the single-porosity modeling approach was used in HYDRUS-1D simulations because the dual-porosity approach required more parameters which were almost non-measurable. Therefore, it was quite difficult to obtain adequately accurate results by direct modeling.

HYDRUS-1D simulated SWC and EC throughout the profiles of 5 and 4 of the first 8 treatments (see Table 2), respectively, better than VS2DTI in the 1st h of the experiments (Fig. 1), whereas it was better than VS2DTI in estimation of SWC and EC in 7 and 5 out of 8 treatments, respectively, in the 2nd h of the experiments (Fig. 2). As HYDRUS-1D had better performance than VS2DTI in estimation of SWC and EC in 7 and 5 of the 8 treatments, respectively, in the 5th h of the experiments (Fig. 3), it simulated SWC and EC better than VS2DTI in 6 and 5 of the treatments, respectively, in the 15th h of the experiments (Fig. 4). Overall even though the models had relativel low performances in estimation of EC, the results clearly showed that HYDRUS-1D was better than VS2DTI in estimation of EC and especially SWC.

The model parameter values used in the modeling studies were presented in Table 3. The first 6 parameters were used in both models, but the 7th parameter in only HYDRUS-1D. The values of the first 2 soil hydraulic function parameters (*θ*_r_, *θ*_s_, *α*, *n*) of van Genuchten (1980) decreased with depth as the sand content of the soil increased, whereas the other 2 parameter values were relatively high in the last 2 layers. *K*_s_ values were high at the surface and bottom depths with high clay and sand contents, respectively, may be due to macropores near the surface and large openings in the sand layers at the bottom. Dispersivity was constant throughout the profiles. Forkutsa et al. (2005) modeled multilayer water flow and solute transport in the rooting zone of irrigated cotton in sandy loam soil in Uzbekistan using HYDRUS-1D. They found *θ*_r_, *θ*_s_, *α*, *n*, *K*_s_, and *l* as 0.043 cm^3^ cm^−3^, 0.427 cm^3^ cm^−3^, 0.021 cm^−1^, 1.470, 0.96 cm h^−1^, and 0.50, respectively, for the top 10 cm, whereas these parameter values were 0.040 cm^3^ cm^−3^, 0.329 cm^3^ cm^−3^, 0.039 cm^−1^, 1.450, 3.42 cm h^−1^, and 0.00, respectively, for the 35–60 cm depth. The parameters *θ*_r_, *θ*_s_, and *K*_s_ had similar values with this study in especially last two sandy layers.

**Table 3:** Model parameters used in the modeling studies

HYDRUS-1D and VS2DTI							HYDRUS-1D
Depth (cm)	*θ*_r_^a^ (cm^3^ cm^−3^)	*θ*_s_ (cm^3^ cm^−3^)	*α* (cm^−1^)	*n*	*K*_s_ (cm h^−1^)	Disp. (cm)	*l*
10	0.19596	0.60826	0.04140	1.38919	12.288	5	−2.1070
20	0.19855	0.56370	0.03634	1.52509	8.965	5	−1.8379
30	0.15437	0.50098	0.03734	1.48646	5.496	5	−1.8102
40	0.10786	0.40453	0.02904	1.54336	9.296	5	−1.4231
50	0.05955	0.31004	0.03286	1.50970	4.657	5	−1.3609
60	0.04920	0.18809	0.04545	1.71304	8.852	5	−0.9714
75	0.03494	0.13564	0.06045	1.54658	9.392	5	−0.6272

*Disp*. dispersivity

^a^ *θ*_r_, *θ*_s_, *α*, *n*, *K*_s_, and *l*: Soil hydraulic function parameters of van Genuchten (1980) and Mualem (1976); *θ*_r_ and *θ*_s_: Residual and saturated SWC, respectively; *α* and *n*: Shape parameters of SWRC; *K*_s_ and *l*: Hydraulic conductivity parameters in Mualem+ (1976) function

The relatively low performances of the models in the simulations of SWC and EC in especially some experimental conditions may be due to the fact that the measurements of SWC and EC using TDR are problematic in especially clayey field soils, thereby complicating the solute transport modeling studies in these soils (Persson et al. 2000). The possible reasons of problems in the measurements may be the result(s) of individual and/or interactive effects of factors like high soil EC, high SWC, high clay content of the soil, soil compaction, and tilting and compacting soil during probe installation (Campbell Scientific Inc 2006). In addition, the 75 cm soil profile had texturally contrasting four layers with different physical and hydraulic properties. This spatial (vertical) complexity in the soil might add some complications on the TDR mesurements of SWC and EC. Moreover, the simulation performance of HYDRUS-1D may be improved by using the dual-porosity modeling approach with the inverse modeling to better determine multi-parameter values (Ritter et al. 2004).

Statistical analyses were done using Tukey test to compare the means of the measured and model (HYDRUS-1D and VS2DTI) results of SWC and EC for 12 treatments and the results were presented in Table 4. The treatments produced 4, 2 and 3 groups for measured and simulated (by HYDRUS-1D and VS2DTI) SWC, whereas they produced 4, 3, and 3 groups for measured and simulated EC, respectively. There were statistically significant differences among the groups in *p* < 0.01 level (Table 4). The division of the treatments into different number of groups indicated that the treatments had different flow and transport characteristics. Therefore, different experimental conditions produced different results with changing degree of non-uniform or preferential flow and transport.

**Table 4:** Comparison of the means of the measured and simulated SWC and EC of the treatments by using Tukey test

Treatment											
Soil type	Soil structure		Initial SWC		Solution application rate					Model	
SCL	Undisturbed	Disturbed	Dry	Wet	Low	Intermediate	High	Parameter	Measured	HYDRUS-1D	VS2DTI
X	X		X		X			SWC	0.274^c^	0.204^a^	0.232^b^
								EC	0.075^ab^	0.106^b^	0.079^b^
X	X		X			X		SWC	0.215^b^	0.192^a^	0.215^b^
								EC	0.060^a^	0.121^c^	0.088^c^
X	X		X				X	SWC	0.177^a^	0.191^a^	0.215^b^
								EC	0.046^a^	0.099^b^	0.074^b^
X		X	X		X			SWC	0.215^ab^	0.200^a^	0.224^bc^
								EC	0.058^a^	0.106^b^	0.081^b^
X		X	X			X		SWC	0.280^b^	0.224^a^	0.238^a^
								EC	0.085^a^	0.099^a^	0.093^b^
X		X	X				X	SWC	0.334^d^	0.234^b^	0.259^c^
								EC	0.108^c^	0.105^b^	0.080^b^
X	X			X	X			SWC	0.318^b^	0.209^a^	0.317^b^
								EC	0.084^b^	0.139^b^	0.129^c^
X	X			X		X		SWC	0.308^c^	0.250^a^	0.270^b^
								EC	0.079^a^	0.099^a^	0.085^a^
X	X			X			X	SWC	0.308^c^	0.212^a^	0.264^b^
								EC	0.081^b^	0.141^b^	0.083^b^
X		X		X	X			SWC	0.377^d^	0.244^a^	0.299^b^
								EC	0.105^b^	0.135^b^	0.102^b^
X		X		X		X		SWC	0.380^d^	0.258^a^	0.296^b^
								EC	0.110^b^	0.135^c^	0.120^c^
X		X		X			X	SWC	0.382^c^	0.318^b^	0.285^a^
								EC	0.115^d^	0.104^b^	0.093^b^

The same letters indicate the same groups and there is no statistically significant difference between these groups in *p* < 0.01 level

*SCL* sandy clay loam, *SWC* soil water content, *EC* electrical conductivity

The means of the solute transport parameters (*v* and *D*) were also compared by the Tukey test and the results were presented in Table 5. The treatments were divided into different groups for both *v* and *D* and there were statistically significant differences among the groups in *p* < 0.01 level. The differences among the treatments in terms of the solute transport parameters also showed that the solute transport was not at least uniform but non-uniform at different scales (Owabor et al. 2010).

**Table 5:** Comparison of the means of the solute transport parameters of the treatments by using Tukey test

Treatment									
Soil type	Soil structure		Initial SWC		Solution application rate			Solute transport parameter	
SCL	Undisturbed	Disturbed	Dry	Wet	Low	Intermediate	High	v (cm h^−1^)*	D (cm^2^ h^−1^)
X	X		X		X			30.24^abc^	198.10^abcd^
X	X		X			X		1.06^a^	1.00^a^
X	X		X				X	1.06^a^	1.00^a^
X		X	X		X			24.98^abc^	3.00^ab^
X		X	X			X		1.06^a^	1.00^a^
X		X	X				X	87.65^d^	357.44^abcd^
X	X			X	X			27.14^abc^	148.22^abcd^
X	X			X		X		9.53^ab^	1.00^a^
X	X			X			X	9.53^ab^	1.00^a^
X		X		X	X			29.08^abc^	153.13^abcd^
X		X		X		X		9.53^ab^	1.00^a^
X		X		X			X	4.09^a^	28.32^abc^

The same letters indicate the same groups and there is no statistically significant difference between these groups in *p* < 0.01 level

*SCL* sandy clay loam, *SWC* soil water conent, *v* pore water velocity, *D* dispersion coefficient

## Conclusions

This paper presented the investigation of the individual and interactive effects of several factors on the extent of preferential flow and transport in a sandy clay loam field soil by using TDR measured SWC and EC, modeling the measured data through HYDRUS-1D and VS2DTI, and comparing the means of the measured and modeled SWC and EC and solute transport parameters of 12 treatments using statistical analyses.

Research results showed that the applied salt-solution moved faster in the undisturbed soil than in the disturbed soil and even passed out the profiles of the undisturbed soil at later times in especially wet initial SWC conditions. Therefore, the effects of soil structure (undisturbed and disturbed) on water flow and solute transport were more distinctive in the wet initial SWC condition. While water and solute moved more rapidly in the wet initial SWC condition than in the dry initial conditions, the effects of dry and wet initial SWC conditions on flow and transport characteristics were more obvious under the disturbed soil conditions. The applied solution moved lower depths in the treatments having higher application rates under constant soil structural and initial SWC conditions, but there seemed to be no obvious differences between the effects of application rates on water and solute movement under different soil structural and initial SWC conditions. The results suggest that the interactive effects on water flow and solute transport necessitate further investigation with more contrast interactions in this sandy clay loam soil.

Overall even though the models had relatively low performances in the estimation of SWC and EC, HYDRUS-1D was better than VS2DTI in estimation of EC and especially SWC. The relatively problematic TDR measurements in especially sandy clay loam field soils in addition to the modeling errors emerged from parameter value determination and fitting procedures may result in the low model performances. However, the model performances may be improved by applying the inverse modeling and using the dual-porosity modeling approach in HYDRUS-1D.

The division of the treatments into different number of groups based on SWC, EC, and solute transport parameters using Tukey test indicated that the treatments had different flow and transport characteristics. In other words, there was varying degree of non-uniform or preferential flow and transport in the soil.
